# Honey and its nutritional and anti-inflammatory value

**DOI:** 10.1186/s12906-020-03170-5

**Published:** 2021-01-14

**Authors:** Yazan Ranneh, Abdah Md Akim, Hasiah Ab. Hamid, Huzwah Khazaai, Abdulmannan Fadel, Zainul Amiruddin Zakaria, Mohammed Albujja, Mohd Fadzelly Abu Bakar

**Affiliations:** 1 Department of Technology and Natural Resources, Faculty of Applied Sciences and Technology, Universiti Tun Hussein Onn Malaysia, 86400 Pagoh, Johor Malaysia; 2 Department of Biomedical Sciences, Faculty of Medicine and Health Sciences, Universiti Putra Malaysia, 43400 UPM Serdang, Selangor Malaysia; 3 Sport and Exercises Sciences School, Faculty of Science, Liverpool John Moores University, Liverpool, UK; 4 Department of Forensic Biology, Faculty of Forensic Sciences, Naif Arab University of Security Sciences, Riyadh, 14812 Saudi Arabia

**Keywords:** Honey, Chronic inflammation, Bioavailability, Bioaccessibility, Bioactive compounds

## Abstract

Inflammation is the main key role in developing chronic diseases including cancer, cardiovascular diseases, diabetes, arthritis, and neurodegenerative diseases which possess a huge challenge for treatment. With massively compelling evidence of the role played by nutritional modulation in preventing inflammation-related diseases, there is a growing interest into the search for natural functional foods with therapeutic and preventive actions. Honey, a nutritional healthy product, is produced mainly by two types of bees: honeybee and stingless bee. Since both types of honey possess distinctive phenolic and flavonoid compounds, there is recently an intensive interest in their biological and clinical actions against inflammation-mediated chronic diseases. This review shed the light specifically on the bioavailability and bioaccessibility of honey polyphenols and highlight their roles in targeting inflammatory pathways in gastrointestinal tract disorders, edema, cancer, metabolic and cardiovascular diseases and gut microbiota.

## Background

Honeybees which are named in Latin as *Apis*, use the collected nectar from plants to produce honey after regurgitation and digestion of nectar. Several biological compounds from honeybees are added during honey formation. Honeybees store honey to be used during winter. Their wings fan the honey to evaporate the water content in nectar to avoid fermentation of honey. Honey has been used to treat a variety of ailments such as gastric disturbance, skin burn and ulcers [1]. Currently, two types of honey are produced globally: traditional *Apis mellifera* honey and stingless bee honey. Honey has been reported to have healthy benefits which are antioxidant [2], anti-proliferative [3], and anti-bacterial [4]. The purpose of this review is to summarize another aspect of the honey’s health benefit which is the anti-inflammatory studies of honey underlying the possible mechanisms involved in the response to honey supplementation on inflammation-mediated chronic diseases as proposed by a wide collection of scientific papers in the literature.

## Honey composition

Honey contains macro and micronutrients which depends basically on various factors: 1) bee type, 2) floral source, and 3) environmental and processing factors. In general, there are approximately 200 compounds in honey such as sugar, protein, enzymes, minerals, vitamins, amino acid, and a wide range of polyphenols. The variety ratio of these compounds results in different color, taste, viscosity, and therapeutic activities of each honey. In this sense, the combination of all these compounds perform synergistically in different aspect of applications [5]. Most of the honeys over the world share 80% of the physical properties and chemical composition. Based on that, various methods have been developed to discriminate the entomological origins of honey and other factors using nuclear magnetic resonance [6]. The previous techniques provide the literature with specific results related to the differences in composition between *Apis mellifera* honey and stingless bee honeys.

### Macronutrients of honey

**Fig. 1:**
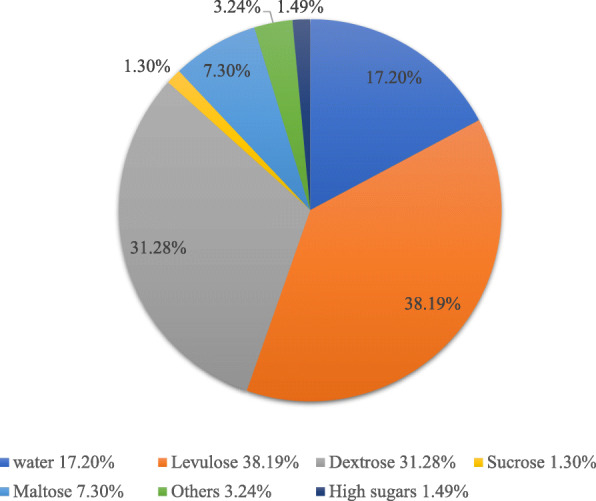
Pie-chart of honey composition demonstrating the percentage of carbohydrates which is almost 80% while water is around 17%. Other components such as enzymes, proteins, vitamins and polyphenols represent 3.24%. The numbers shown in pie-chart are varied in each type of honey

The macronutrient composition of honey as shown in Fig. 1 represents an interesting source of carbohydrates which is the main core of honey and support the anti-spoilage properties. The carbohydrate ratio is ranged from 60 to 95% of its dry weight including mono-, di- and tri-saccharides where floral type is a key factor in modulating this ratio [7]. More than 20 types of carbohydrates have been identified in honeys samples from different part of the world [8]. The principal carbohydrate existed is fructose followed by glucose with 28–40% and 20–35%, respectively, while the disaccharide and trisaccharide concentration are around 5 and 1%, respectively [9]. The most identified disaccharides are maltose, maltulose, turanose, sucrose, nigerose whereas a few trisaccharide such as erlose, centose, isomaltotrios, panose, psopanose and ketose are found in small amount [10].

The protein content of honey is roughly ranged from 0.2–0.5% in forms of enzymes and free amino acids. Generally, the total amount of free amino acid in honey range approximately between 10 and 200 mg/100 g honey and proline contribute with 50% of total amino acid [11]. G-aminobutyric acid and ornithine have been identified in honey samples in addition to b-alanine and a-alanine [12]. While the main source of protein and amino acid is pollen, honeybee contribute to modifying this profile through regurgitation. Defensin-1 protein has been found in bee hemolymph and subsequently in different honey samples except Manuka honey [13]. Lipids quantity in most of honey samples is negligible about 0.002%. Plants and wax take part primarily in the exitance of various lipids compounds in form of acids such as palmitic, oleic, miristic, and linoleic acid [14].

### Micronutrients of honey

The minerals and vitamins profile in honey are varied according to floral type and geographic origin, representing from 0.2–0.5% of honey dry weight. Although essential minerals and elements are trace in honey, human body need them to perform the several biological actions perfectly [15]. Several studies have been conducted to determine the minerals content in honey samples over the world in order to reveal the existence of contaminant minerals [16, 17]. Potassium and sodium constitute mostly 80% of total minerals while iron, copper and manganese are rare in quantity. Moreover, trace elements have been recruited recently for identification purposes of different unifloral honeys [18]. To illustrate more, the presence mixture of potassium, cadmium, and nickel was implemented as a strong indicator to distinguish honeydew honey while barium and lead were specific components of rape honeys [19]. With respect to vitamins, one study has found thiamin, riboflavin, pyridoxin, niacin and ascorbic acid in several honey samples, however, their amount do not correspond with the recommended daily intake of human [20]. The lipids and fat-soluble vitamins content in honey are hard to detect.

### Enzymes in honey

Unlike other sweetening agents, honey contain various active enzymes that play key role in its biological function. The source of these enzymes is probably from nectar, bee, or micro-organisms in honey [21]. Invertase, glucose oxidase, and diastase are the main enzymes in honey. Although invertase is involved in catalyzing sucrose into its constituent in honey, small amount of sucrose is still present in the final stage of honey ripening [22]. Diastase’s function is to breakdown the chemical bonds in starch and mainly in maltose, even though starch is not detected in any honey samples. Thus, the original function of diastase in honey remain unclear, however, few methods have developed to measure diastase as a quality indicator of honey, where the quality of honey is positively proportional to diastase amount [7, 22]. Glucose oxidase is one of carbohydrate metabolizing enzymes. The nativity of glucose oxidase is the pharyngeal glands of bee and thus its amount is varied. Glucose oxidase breakdown glucose into gluconic acid, which is one of the importance honey acids, and hydrogen peroxidase. The presence of glucose oxidase has prevented the microbial growth in honey [23]. However, glucose oxidase could be affected by many factors which explain, in part, of its absence in various types of honey samples [13].

### Bioaccessibility and bioavailability of honey polyphenols

The presence of polyphenol in honey is believed to be from the plant’s nectar while polyphenols quality and quantity depend on the geographical region, floral source, climatic conditions, and bee type [7]. Therefore, one study has suggested that polyphenol profile of honey could be as a floral marker to verify the botanical origin [24]. The attempts to identify the phenolic profile of honey samples have been noticed in many food analytical studies [2, 25]. Most of these studies have utilized mainly HPLC-UV and HPLC-DAD for quantification and identification purposes with various modified methods. However, LC-MS/MS has been concluded to be more accurate than LC-MS, while GC-MS has been recruited mainly to identify the volatile compounds [26]. Furthermore, the previous mentioned technologies contribute to creating phenolic profiles for many types of honey despite of the complex chemical structure of honey. Thus, developing an optimized method to identify and quantify polyphenols in honey is still required.

The existence of one or more phenolic compounds in honey samples have been claimed as phytochemical markers for the floral origin. Founded free in honey samples, phenolic acids such as *p*-coumaric, gallic acid, caffeic acid and ferulic acid were commonly identified in different floral type of honey [27–29]. However, depending on few phenolic and flavonoid compounds is not sufficient to determine the multi-floral origin of honeys where the same compounds could be existed. To explain more, the verification of honey floral is undermined by the concentration and content among polyphenols compounds [30]. Considered as biomarkers for rosemary and sunflower honeys, quercetin and kaempferol have been found in high levels of pumpkin, rapeseed, and melon honeys [31]. Thereby, adding other chemical factors to verify closely the geographical origin of honey remain the proper option.

The phenolic content of honey has been correlated with antioxidant activities in many published researches [32]. In addition, honey’s phenolic profile reflects the bees’ type, botanical origin, season, and the region. One study compares the phenolic content between the common honeybee and stingless bee honey. The total phenolic content of Malaysian stingless bee honey was approximately 235 mg GAE equivalent 100 mg compared to Tualang honey 183 mg GAE equivalent 100 mg, while the flavonoid content of stingless bee honey was 100 mg CE equivalent 100 mg. In the same study, the antioxidant results of stingless bee honey were significantly higher than the normal honey [2]. Likewise, the honey of stingless bee from Plebia spp. species has higher phenolic content than Apis spp. 106.01 ± 9.85 mg GAE equivalent/100 g compared to Apis spp. 92.34 ± 13.55 mg GA acid equivalent/100 g, respectively [33]. Furthermore, honey produced from Meliponini, a stingless bee species, has the highest radical scavenging activity against ABTS cation due to the high levels of phenolic and flavonoid contents. Contributing in proinflammatory state, ROS has been inhibited after treating with honey extract from stingless bee [34]. In the same study, the author concluded that the stingless bee honey-rich in polyphenol had suppressed the infiltration of leukocyte through downregulating myeloperoxidase and reduced the ear edema [34]. Previous studies-related to the medicinal value of stingless bee honey indicate that there is a deep limitation of reported research. The potential therapeutic value of stingless bee honey is needed to be widely explored against inflammatory and oxidative stress related disease. Probably, the phenolic compounds in stingless bee honey have high pharmacokinetics and react synergistically in preventing or treating disease.

Since polyphenol compounds have retained a great attention by scientific communities as preventive agents against degenerative and chronic inflammatory diseases [35], the main therapeutic activities of honey are attributed to its content of polyphenols because they are the most abundant phytochemcials [2, 36]. These activities are controlled by the bioaccessibility and bioavailability of polyphenols in human body. In terms of bioaccessibility, polyphenols which is released from the food matrix or absorbed by small intestine through mechanical and biochemical breakdown are potentially bioavailable and bioactive [37]. However, the molecular interaction of polyphenols with other food compounds during digestion process may decrease or increase the bioaccessibility [38, 39]. For example, the bioaccessibility of anthocyanin has been varied when testing its release from different foods (whole berries, berry juice, wine, jam, powders) [40]. The existence of dietary fibers in food matrix play a major role in releasing polyphenols for absorption into gastrointestinal tract [37]. Few clinical trials illustrated that less than 10% of polyphenols (aglycones and glucoside conjugate) are being absorbed in the upper gastrointestinal tract due to the effect of food matrix, while the rest of phenolic compounds undergo to microfloral metabolization at which bioactive metabolites cross colonic mucosa to plasma [37, 41, 42]. At the same time, it is apparent that polyphenols may have high affinity to protein and fibers, especially if all the previous components are present together. Thus, the presence of polyphenol in a food matrix with negligible amounts of dietary fibers, protein and lipids could reduce the molecular interactions and may increase the bioaccessibility and bioavailability ratio in honey. In this sense, the phenolic content and antioxidant activity of Manuka honey were not altered after subjecting to in-vitro digestion process compared with other commercial honey samples [43]. In addition to that, 1-3 mg/ml of post- and pre-digested Manuka honey has significantly protective ability against hydrogen peroxide induced DNA damage in Caco-2 cell line. This demonstrate that polyphenols in Manuka honey are not affected by any molecular changes due to digestion process and thus, they may have high bioaccessibility ratio [43]. In another study, adding floral and pine honey to brewed coffee significantly increased the levels of phenolic content and antioxidant activities after simulating digestion process in vitro. The possibilities of using honey as a natural sweetener synergize the phenolic content of coffee and consequently, leading to high bioavailability [44]. After mimicking the gastric and intestinal digestion, the phenolic content and antioxidant activities of Turkish honeybee pollen have been reduced which could probably due to either the interaction of polyphenols and protein honeybee pollen matrix or the difference in pH levels which influence the bioactivity of phenolic compounds. Therefore, maximizing the effect of honeybee pollen could be achieved through encapsulation [45]. However, bractinga honeydew honey has shown different results in terms of phenolic content and antioxidant capacities where in vitro duodenal digestion decreases the antioxidant stability and increase the flavonoid content. The bioaccessible fraction was increased from 112% after gastric ingestion to become 174% after duodenal digestion [46]. Overall, the previous studies have used in vitro model to mimic digestion process in which honey polyphenols stability have been examined and demonstrated potential bioaccessibility and bioavailability. Possibly, this stability observed in vitro may not be the same as in vivo due to differences in conditions and concentrations of honey.

Determining the best phenolic compounds in terms of absorption and producing bioactive metabolites is still occupying the main objective in bioavailability studies through which the biological actions of polyphenols within targeted tissues could be revealed and classified. As a result of that, bioavailability studies have measured the phenolic compounds concentrations in plasma and urinary execration among subjects supplemented with polyphenols as pure compound, plant extract or whole food [39, 47]. While polyphenols-derived metabolites, affected by the gastric and hepatic processes, could be inactive, detecting these metabolites in plasma and urine requires a rigorous technique. Also, there is a lacking research with respect to the bioavailability of polyphenols in human. Thus, these challenges could attract researchers to investigate the pharmacokinetics and pharmacodynamics of honey as rich food in polyphenol. Moreover, human interventions trials with honey seem to be overpowered. The phenolic acid and flavonoid in honey are varied with different bioavailability. In general, phenolic acids ratio are used to be higher than flavonoid in honey. At the same time, phenolic acid classes; hydroxycinnamic derivatives and hydroxybenzoic derivatives are well absorbed in human body as they are in aglycon form [47]. Gallic, caffeic, *p*-coumaric, and sinapic acid have been proven to be well absorbed in the upper part of gastro-intestinal system despite of the differences in kinetic efficacy [37]. Recently, it has been indicated that stomach could have an active absorption site for the above-mentioned phenolic acids [48]. The mechanism of absorption is mediated through monocarboxylic acid transporter and paracellular diffusion. On the other hand, flavonoid absorption in small intestine is considered as a complex process where flavonoids, in forms of glycoside, require hydrolysis process to convert into aglycones. This hydrolytic process is performed through two enzymes: 1) lactase phlorizin hydrolase acting in the epithelial cells, 2) cytosolic *β*-glucosidase in the enterocyte. Then, aglycones can be absorbed by the intestinal cells namely, enterocytes crossing to the blood stream [39, 41, 47]. The existence of glycosidase enzyme in bee salivary glands contribute essentially to hydrolyzing glycosylated polyphenols into aglycones form. Therefore, honey polyphenols which are in aglycones forms, could have more potential bioavailability than other foods [49]. However, a direct investigation on the bioavailability of honey polyphenols either in vivo or in human has not been conducted yet. In contrast, administrating natural honey as a dietary supplement in various animal and clinical trials have either improved or ameliorated the pathological status of studied subjects [1, 50]. Indeed, consuming 1.5 g of honey per kilogram of human body has elevated the antioxidant status in healthy individuals comparing with those who have consumed the same amount of corn syrup [51]. Although the previous study may promote the pharmacokinetics of honey polyphenols, the possible action of honey polyphenols in improving the total antioxidant could be indirectly throughout supporting the endogenous antioxidant system. A randomized clinical trial has revealed the supportive effect of Tualang honey on glutathione peroxidase, superoxide dismutase and catalase activities in chronic smokers’ subjects [52]. Although some individuals have differences in the uptake and metabolism of phytochemical due to polymorphism, honey intervention trials demonstrate that the variability in the level of honey polyphenols absorption could be low [53]. Hence, determining the bioavailability of honey polyphenols in tissues is crucial to confirm the percentage of absorption. The conjugation reaction of polyphenols in hepatic tissues to detoxify potential toxicity and increase hydrophilicity is thought to impair the bioactivities of polyphenols through glucorination, methylation, and sulfation. In blood circulating, polyphenols metabolites concentration has been found mostly in nanomolar and micromolar range [39, 54]. Also, determining pharmacokinetics of phenolic compounds have been selectively performed on few types of phenolic compounds, due to the large number of polyphenols which are eight thousand. Despite of being proven to have a low plasma concentration of some phenolic acid and flavonoid, the other phenolic compounds could be different. It is noteworthy that the physiochemical profile of polyphenols (molecular size, basic structure, degree of polymerization, solubility, conjugation with other compounds) contribute essentially in absorption and metabolism process and consequently, affect their biomolecular interaction [39]. In terms of honey, conceivable hypothetical explanations could interpret the positive effect on human intervention studies i) honey polyphenols absorbed slowly through gastrointestinal tract, ii) the simplicity of honey structure, iii) polyphenols metabolized completely in the tissues, iv) unknown phenolic compounds and/or their metabolites in honey could have high bioavailability and penetrate cells with special receptors, v) the absence of anti-nutrients compounds. In conclusion, bioaccessibility and bioavailability of honey polyphenols are affected by several factors such as floral origin, food matrix, gastrointestinal absorption, liver and intestinal metabolic process, binding to albumin, tissue accumulation and execration. Hence, assimilating these factors and attributing them to the healthy pleiotropic effects are a big challenge. Additionally, reconstructing instrumental analytical equipment with advanced selectivity and sensitivity is a required step to understand the pharmacokinetics and pharmacodynamics of honey polyphenols. But at the same time, the empirical evidence of honey intervention on direct interaction with DNA and gene expression have been revealed [55, 56]. The study to identify potential molecular mechanisms involved in the cellular interactions by various types of honey polyphenols with its relevant gene expression profiles should be extensively made.

## Inflammation

Inflammation is the natural innate response of immunity system to pathogens where various cellular and humoral immune are developed [57]. At the same time, oxidative stress is present when the balance remains in favor of the overproduction of free radicals over the antioxidant components. Inflammation and oxidative stress are associated with each other via multiple signaling pathways [58, 59]. Reactive oxygen species (ROS) produced from mitochondria, trigger various transcription factors (NF-κB, ERK, P38, JNK, MAPK) related to producing pro-inflammatory cytokines and mediators. Likewise, few cytokines namely, TNF-α and IL-1β can induce the production of ROS from mitochondria [60, 61]. This correlation relationship between ROS and pro-inflammatory cytokines results in metabolic and cellular modifications [62]. In other words, the onset of uncontrolled inflammatory process along with the presence of oxidative stress plays a key role in the pathophysiological incidences of chronic disorders such as, psychiatric, cardiovascular, traumatic, metabolic, and autoimmune maladies. Recently, there is growing evidence indicating that honey could have inhibitory effect on chronic inflammation, oxidative stress and on their relative gene expression [63]. Also, a group of transcription factors such as Nrf2, ERK1/2, NF-κB, c-Jun and AP-1 have been implicated. These transcription factors are responsible for numerous biological process, primarily production of antioxidant compounds and inflammatory cytokines [64–66]. Synthetic drugs have been developed as agonists to ameliorate cancer, ageing process, and CVDs [67, 68]. It has been suggested that honey compounds including polyphenols could act as agonists for NF-κB receptors and Toll-Like 4 receptor that are involved in the initiation of inflammation and oxidative stress.

## Potential health benefits of honey against inflammation

**Fig. 2:**
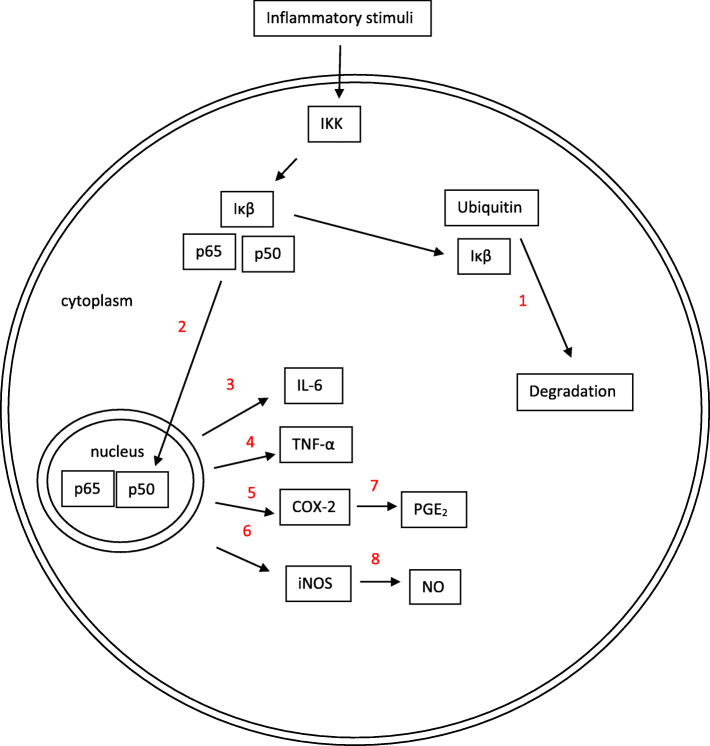
Schematic diagram of the effect of honey on the cytokines in the inflammatory response upon stimuli in a cell. No 1 until 8 in red denotes the inhibition site of honey. Edited from (Hussein, Mohd Yusoff, Makpol, & Mohd Yusof, [55])

Innate immunity relies strongly on inflammation which is defined generally as the natural response to cellular injury. The biological changes are 1) increased blood flow and capillary distillation, 2) leukocyte infiltration, and 3) releasing localized chemoattractants to recruiter immunity cells. The main objectives of these changes are to eliminate the pathogen agents and repair the damage tissue [69]. However, non-resolving inflammation as result of chronic bacterial infection (LPS infusion) or obesity or aging paves the onset of low-grade chronic inflammation which subsequently develop various chronic diseases. In this context, supporting the consumption of natural products in favor of resolving inflammation and supporting hemostasis is the main reason for examining these products on numerous trials. Honey has been suggested as an immune-modulatory agent with dual role: (1) anti-inflammatory activities through downregulating the inflammatory transcription factors (NF-κB and MAPK) and/or suppressing the production of pro-inflammatory cytokines, and (2) stimulate the production of inflammatory mediators such as prostaglandin E_2_ (PGE_2_) and cyclooxygenase-2 (COX-2) [68]. Various models of inflammation treated with honey have been studied to explain the honey bioactivities towards inflammation (Table 1). Figure 2 represent the schematic diagram on the inhibition of honey on various reactions in the inflammatory process.

**Table 1:** In vivo and in vitro studies on the effect of different types of honey

Effect of honey in in vivo study	
A combination of insulin and honey	This combination inhibited neuronal cell death in different hippocampal areas of streptozotocin-induced diabetic rats [56].
Alimento Supervis and Alimento Mieleucalipto, which are derived from chestnut honey	Oral pretreatment (2 g/kg), once daily for 7 consecutive days, prevented indomethacin-induced gastric lesions in rats by reducing the ulcer index, microvascular permeability, and myeloperoxidase activity of the stomach [70].
Tualang honey (TH)	TH has almost the equal effects when compared with the conventional treatment in treating alkali injury on rabbit’s eye based on no significant difference in the level of total antioxidant status as well as lipid peroxidation products in aqueous humour, vitreous humour and serum between honey treated and the conventional treated group [71].
Manuka honey (MH)	MH significantly increase enzymatic (GPx and SOD), nonenzymatic (GSH) antioxidants levels and anti-inflammatory cytokine IL-10 levels. It normalized cell cycle distribution and significantly lowered apoptosis in gastric mucosa [72].
Honey	Intrarectal honey administration is as effective as prednisolone treatment in an inflammatory model of colitis [73].
Honey	Honey prevented gastric mucosal lesions induced by ethanol, indomethacin, and acidified ASA. The protection was almost total when using ethanol or acidified ASA as a damaging agent; whereas protection against indomethacin was moderate [74].
Honey	Honey significantly increased CAT, GR, TAS, TGSH, GSH and GSH:GSSG ratio and significantly reduced activities of SOD and GPx, MDA levels and FPG in diabetic rats [75].
*Melipona marginata* honey (MMH)	MMH reduced ear edema and reactive oxygen species production. It also decreased the myeloperoxidase activity [34].
Honey	Honey suppressed the phosphorylation of NFkB in cisplatin-induced kidney dysfunction [76].
Honey	There was almost 100% protection against gastric damage with the highest dose (5 g/kg) of honey used. However, there was only partial protection (58%) against ethanol-induced gastric lesions [77].
Honey	Honey significantly increased high density lipoprotein (HDL) cholesterol while it significantly reduced hyperglycemia, triglycerides (TGs), very low-density lipoprotein (VLDL) cholesterol, non-HDL cholesterol, coronary risk index (CRI) and cardiovascular risk index (CVRI). It also significantly reduced TGs and VLDL cholesterol [78].
Tualang honey (TH)	TH significantly reduced blood glucose levels compared to the diabetic control rats’ group. It significantly reduced elevated MDA levels and restored SOD and CAT activities [79].
Gelam honey (GH)	GH gave its anti-inflammatory effects by reducing the rat paw edema size and inhibiting the production of proinflammatory mediators NO, PGE_2_, TNF-*α*, and IL-6 in plasma, and suppress the expression of iNOS, COX-2, TNF-*α*, and IL-6 in paw tissue [80].
Gelam honey (GH)	GH exhibited its inhibitory effects by attenuating NF-kB translocation to the nucleus and inhibiting IkBa degradation, with subsequent decrease of inflammatory mediators COX-2 and TNF-a [55]
Mad honey or rhododendron honey	It significantly lowered MDA levels and TNF-α and MMP-9 expression, and increased antioxidant enzyme activities and IL-10 expression in male Wistar albino rats with streptozotocin-induced diabetes mellitus [81]
Honeybee venom (HBV)	Low doses of HBV have been shown to treat RA with anti-inflammatory and antioxidant effects, by preventing DNA damage. After low-doses of HBV treatment IL-1β, IL-6, TNF-α, TGF-β1, TOS, OSI, MPO and MNL-DNA damage levels significantly decreased according to the PC, while IFN-γ and TAS levels increased [82].
Trihoney (a combination of three types of natural honey namely: Trigona, mellifera, and Dorsata)	It significantly lowered serum IL-1β and IL-6 compared to the high cholesterol diet group of male New Zealand white rabbits and resulted in significant reduction of serum TNF-α compared to high cholesterol diet group [83].
Honey	It inhibited edema and pain in inflammatory tissues as well as showing potent inhibitory activities against NO and PGE_2_ in both non-immune inflammatory and nociceptive model, and lipopolysaccharide (LPS) in the immune inflammatory model [84].
Manuka Honey (MH)	In inflammatory model of colitis, oral administration of MH (5 g/kg) and combination with sulfasalazine (360 mg/kg) with MH (5 g/kg) significantly reduced the colonic inflammation [85].
Honey	Honey-treated in dextran sodium sulphate-induced colitis group significantly exhibited the down-regulation of oxidative, inflammatory, and apoptotic markers (interleukin-1β and − 6, superoxide dismutase, reduced glutathione, tumour necrosis factor-α, NO synthase, caspase-3, CD34, Ki67, S100, c-kit, and neuron-specific enolase) [86].
Effect of honey in in vitro study	
A combination of Gelam honey and ginger extract	modulate Ras/ERK and PI3K/AKT pathway genes, upregulate caspase 9 and IκB genes accompanied by downregulation of the KRAS, ERK, AKT, Bcl-xL, NFkB (p65) genes in a synergistic manner in colon cancer HT29 cells [87].
A combination of Bee honey (BH) and *Nigella sativa* (NS)	significantly decrease in both the number of viable HepG2 cells and the levels of nitric oxide on one hand, but improvement of the total antioxidant status and caspase-3 activity [88].
Manuka honey (MH)	MH protected mitochondrial functionality, promoted cell proliferation, and activated the AMPK/Nrf2/ARE signaling pathway, expression of the antioxidant enzymes SOD and CAT in Primary Human Dermal Fibroblasts (HDFa) [89].
Stingless bee honey	Two out of eight stingless bee honey types (Meliponinae) from southern Brazil increase the secretion of anti-inflammatory cytokine (interleukin-10) in RAW 264.7 macrophages [90].
Kelulut honey	at a concentration of 1%; v/v significantly inhibited nitric oxide (NO) production in LPS-induced RAW 264.7 cells compared to control cells [91].
Tualang honey	inhibited UVB-induced DNA damage, and enhanced repair of UVB-mediated formation of cyclobutane pyrimidine dimers and 8-oxo-7,8-dihydro-2′-deoxyguanosine, inhibited UVB-induced nuclear translocation of NF-κB and degradation of IκBα in murine keratinocyte cell line, inhibited UVB-induced inflammatory cytokines and inducible nitric oxide synthase protein expression, inhibited UVB-induced COX-2 expression and PGE2 production [92].
Manuka honey (MH)	MH protected mouse RAW-264.7 macrophages against LPS-induced inflammation, by improving viability, promoting proliferation, reducing apoptosis, and enhancing energetic metabolism. These effects were linked to the capacity of MH in modulating the expression of several proteins involved in apoptosis, inflammation, metabolism, and mitochondrial biogenesis, such as caspase 3, p38, pErk1/2, AMPK, SIRT1 and PGC1α [93].
Manuka honey (MH)	MH inhibited LPS induced ROS and nitrite accumulation. It suppressed TNF-α, IL-1β and IL-6, and iNOS [94].
Honey	IL-6 secretion was remarkably reduced by all honey varieties in a comparable level indicating the potential anti-inflammatory property of arid region honey [95].
Greek thyme honey-derived monoterpene	significant apoptotic activity in PC-3 cells (prostate cancer cells), mediated, at least in part, through reduction of NF-κB activity and IL-6 secretion [96].
Honey ethyl acetate extract and honey methanol extract	The highest inhibition percentages of NO production in RAW264.7 cells treated with honey extracts after LPS and IFN-*γ* stimulation were 80% (4.3 μmol/L of NO) and 40% (16 *μ*mol/L) for honey ethyl acetate extract and honey methanol extract (100 μg/mL), respectively [97].
Honey bee larva powder	exhibits anti-inflammatory activity by decreasing the production of nitric oxide (NO) and the cytokine level of interleukin-6 (IL-6) in lipopolysaccharide (LPS)-stimulated RAW 264.7 macrophage [98].
Honey bee (*Apis mellifera*) venom	inhibited lipid accumulation and C/EBPα and PPARγ gene expression during intermediate and late 3 T3-L1 cell differentiation. It also suppressed gene expression of pro-inflammatory cytokines (COX-2, iNOS, MCP-1, TNF-α, IL-1β and IL-6) in LPS-stimulated macrophages, and in co-culture of 3 T3-L1 adipocytes and RAW264.7 macrophages [99].
Honeys produced from *Echium plantagineum* L	decrease NO levels in lipopolysaccharide-stimulated murine macrophage-like cells (RAW 264.7) up to 40% at concentrations of 0.25 mg/mL [100].
Kelulut honey (KH)	KH exerted anti-inflammatory activity in LPS-induced RAW 264.7 cells, where a significant reduction in NO levels was observed at 1% compared to the untreated cells (LPS-induced RAW 264.7 cells) [91].
Honey from different floral sources, including *Bidens pilosa*, *Dimocarpus longan*, *Litchi chinensis*, *Citrus maxima*, and Aglaia formosana.	Honey from Bidens pilosa had significantly greater scavenging activities for 1,1-diphenyl-2-picrylhydrazyl (DPPH·), more reducing power, higher antibacterial activity against gram-positive and gram-negative bacteria compared to others. However, *B. pilosa* honey showed little inhibitory activity against IL-8 secretion, compared to other honeys [101].
Honey proteins	Honey proteins/peptides fractionated by size exclusion chromatography into five peaks with molecular masses in the range of 2–450 kDa inhibit reactive oxygen species production in zymosan-activated human neutrophils and murine macrophages, significantly suppressed the nitric oxide production by LPS-activated murine macrophages, inhibited the phagocytosis latex bead macrophages, did not affect the production of IL-1β; however, TNF-α production was significantly suppressed [102].
Manuka honey (MH)	MH (0.5%) significantly increases the release of CXCL8/IL-8, CCL2/MCP-1, CCL4/MIP-1β, CCL20/MIP-3α, IL-4, IL-1ra, and FGF-13 while reducing proteinase 3 release in the anti-inflammatory-stimulated models. However, higher dose of MH (3%) significantly increased the release of TNF-α and CXCL8/IL-8 while reducing the release of all other analytes [103].
Tesco and Manuka honeys	Both honeys gave significant protective effect against H_2_O_2_-induced DNA damage in Caco-2 cells, following digestion [43].
Bracatinga (Mimosa scabrella Bentham) honeydew honeys	It reduced the nitric oxide secretion as well as the inflammatory mediators: tumor necrosis factor-alpha, interleukin-6, monocyte chemoattractant protein 1, interleukin-12p70, interferon-gamma and interleukin-10 [104].
Honeys (Manuka, pasture, and jelly bush)	All honeys significantly increased the TNF-α, IL-1β and IL-6 release from MM6 cells (and human monocytes) when compared with untreated and artificial-honey-treated cells. Jelly bush honey significantly induced the maximal release of each cytokine compared with manuka, pasture or artificial honeys. The effect of honey on wound healing may in part be related to the stimulation of inflammatory cytokines from monocytic cells [105].
Manuka honey (MH)	A 5.8-kDa component in MH stimulated the production of TNF-α via TLR4 which could improve wound healing [106].

### Inflammatory disorders of the gastrointestinal tract

Gastrointestinal tract depends on various integrated factors that work coherently to maintain the hemostasis of intestinal barrier. These factors include mucus producing-Goblet cells, antimicrobial peptide-providing Paneth cells and commensal microflora [107]. The disturbance of these integrated factors by burns, trauma, or systemic stress dysfunction the gastrointestinal barrier allowing bacterial endotoxin to translocate and initiate inflammation [108]. Recently, it has been proposed that the occurrence of inflammatory gastrointestinal tract disorders have been strongly associated with systemic inflammatory response leading to multi organ dysfunction and ended up with fatality [109]. Few scientific reports have examined honey intake against inflammation in gastrointestinal tract disorders models. For example, ulcerative colitis is considered as one of the chronic inflammatory gastrointestinal diseases affecting 5–10 per 100,000 [110]. However, supplementing natural Egyptian honey (1 g/rat) in dextran sodium sulfate-induced ulcerative colitis in Wister rats has significantly reduced IL-1β and IL-6 in the serum while TNF-α, inducible nitric oxide synthase, caspase-3 have been downregulated in the colonic tissues [86]. Similarly, Turkish natural honey treatment for seven days was noticed to significantly reduce the macroscopic and microscopic lesions in trinitrobenzene-induced ulcerative colitis in Wister rat compared to synthetic drugs supplementation. Surprisingly, nitric oxide was not remarkably reduced through the same period suggesting that resolving the inflammatory process in the colonic tissues were not associated with modulating inflammatory cytokines [73]. Likewise, chronic gastric ulcer in Wister rats feed with 2.5 g/kg of Manuka honey has been alleviated through promoting anti-inflammatory cytokine IL-10 and minimizing pro-inflammatory cytokines TNF-α, IL-1β and IL-6 [72]. The preventive activities of honey against 3% acetic acid-induced colitis in rodents have been also noted with high dose (5 g/kg). This prevention of colonic inflammation accounted for the reduction of systemic pro-inflammatory cytokines. Using the same above-mentioned dose (5 g/kg) of Manuka honey, the scores of ulcerative colitis were reduced [85]. The reduction of inflammatory state after consuming Italian chestnut honey was studied in indomethacin-induced gastric in Sprague dawley rats. Two doses of chestnut honey (1.2 g/kg and 2 g/kg) consumed for seven days had decreased similarly the ulcer lesions, microvascular permeability, and myeloperoxidase activity of stomach throughout enhancing the scavenging performance of free radicals [70]. In fact, although gastro-intestinal tract pathology retains high potential of developing systemic inflammation through allowing the toxic component of gut lumen namely, free fatty acids, inflammatory products of phospholipase A2, proinflammatory cytokines and damage-associated molecular substrates, to pass through portal vein and mesenteric lymph, the above-mentioned studies have assented that honey bioactive compounds contribute positively and significantly towards gastrointestinal tract homeostasis. However, revealing the molecular mechanisms of honey bioactive compounds in gastrointestinal tract disorders is still required.

### Inflammatory edema

The inflammatory response of human tissue is associated usually with the presence of edema specially if the inflamed tissue targets the skin. The accumulation of fluid and white blood cells in the injured area are caused by the secretion of inflammatory cytokines into the bloodstream. In this context, few studies have tested the anti-inflammatory activity of honey against inflammatory edema in skin. The most common inflammatory skin model used is carrageenan-induced paw edema in rodent [111]. Feeding rats with Gelam honey (2 g/kg) for seven days have attenuates the thickness of carrageenan-induced paw edema along with reducing pro-inflammatory cytokines (TNF-α, IL-6, NO, COX-2, PGE_2_) comparing with indomethacin [80]. The same research team has repeated the study to conclude that Gelam honey effect negatively on transcription factor NF-κB translocation into nucleus where IκBα degradation is inhibited [55]. Additionally, injecting Gelam honey (800 mg/kg) intraperitonially into rats challenged with carrageenan and lipopolysaccharide suppressed the production of NO and PGE_2_ leading to a decrement in paw edema and pain [84]. The previous findings emphasize the evidence of anti-inflammatory properties in honey against acute inflammation models. These preventive activities of honey have been tested in two different routes of administration (GI and intraperitoneally) and in different doses.

### Cancer associated with inflammation

Chronic inflammation conditions are associated with elevating various pro-inflammatory cytokines and oxidizing agents at which mutagenicity, toxicity and destabilizing DNA lesions are formed and accumulated leading to increased cancer risk [59]. Thus, measuring pro-inflammatory cytokines along with ROS have been documented in various trials aim to develop promising natural agent to combat cancer [112]. In this context, few oncogenic clinical trials have evaluated the inflammatory damage after honey application and/or consumption in cancer subjects. Oral mucositis which is one of complications in advanced cancer stage initiate an Iranian research team to perform a double-blind randomized clinical trial for three years continuously (2011–2013). Seventy-five subjects from Baqiyatallah University Hospital in Tehran, diagnosed with oral mucositis were enrolled and divided into three groups treated with either steroid, honey, coffee and honey. The level of mucositis was reduced significantly after consuming honey (300 g) every three hours for one week [113]. The previous findings were comparable with another clinical trial where the recovery time of cancer patients diagnosed with chemotherapy-induced oral mucositis was remarkably decreased after topically applying honey (15 g/kg) to the affected area of oral cavity. The previous findings have concluded the attenuation effect of consuming honey on cancer patient through measuring the grade of mucositis [114]. However, applying manuka honey to alleviate the radiation-induced mucositis in neck and head cancer patients was not statistically significant comparing with control group [115]. The differences in honeys type and methodology could account for the varied results among the previous clinical trials. Recently, a meta-analysis study has concluded that the risk reduction of mucositis in cancer patients was almost 80% despite of the variation in methodology among the previous clinical trials which increase the necessity of conducting further studies to confirm the published results with more concern on honey dosage and the way of intervention [116]. Worthy to be mentioned, evaluating the anti-cancerous effect of honey in the above-mentioned trials relied on the clinical observation of the inflammatory damage caused by cancer and/or chemotherapy without determining the inflammatory cytokines in the sera. Thus, the mechanistic actions of honey against cancer still require further investigation on the molecular and genetic levels.

Since chronic infusion of inflammatory cytokine along with ROS are released from activated immune cells, angiogenesis and stroma proliferation induce cancer through mutations [117]. One epidemiological study has reported a reduction of colorectal, prostate, and ovarian cancer incidence among subjects taking non-steroidal anti-inflammatory drugs (NSAIDs) [118]. Further researches on cancer and inflammation revealed that MAPK and NF-κB pathways, which are necessary for inflammatory response, are imbalanced during oncogenesis [58]. Based on that, few inflammatory cytokines and related transcription factors have been determined in multiple cancer models (in vitro and in vivo) after honey treatment [119]. Gelam honey in conjunction with ginger crude extract has shown a chemopreventive ability against colon cancer cell line (HT-29) through modulating ERK/RAS pathways which contribute mainly in tumorigenesis [87]. While nitric oxide elevation has been documented in cancer patients, the preventive efficiency of Egyptian honey against nitric oxide released from hepatocellular carcinoma has been significant comparing with non-treated cells [88]. On the other hand, IL-6 has been up-regulated in different types of malignancy but using serial concentrations of Arabic honey reduce the levels of IL-6 in prostate cancer cell line [95]. The previous findings were in consistent with Kassi and his colleagues, where monoterpene, a special phenolic component in thyme honey, had shown an apoptotic activity mediated by suppressing NF-κB activity and IL-6 secretion in prostate cancer cell line [96]. Thus, the prohibitory effect of honey against pro-inflammatory cytokines in cancer is mediated by NF-κB which could have a promising approach in decreasing the change of developing chronic inflammation. Although honey has a potential antioxidant activity, the activity of Nrf2, which is a transcription factor responsible for antioxidant enzymes, has not been determined.

### Metabolic and cardiovascular diseases associated with inflammation

Low-grade inflammation has been strongly associated with several metabolic changes including, atherogenic dyslipidemia, hypertension, insulin resistance, and abdominal obesity [120]. The previous metabolic changes have been noted in most individuals associated with cardiovascular disease. This study concluded that consuming 75 g of natural honey cause a reduction in LDL-cholesterol. The reason behind this action is probably due to high capacity of honey in free radicals scavenging [121]. Therefore, honey, as it has anti-inflammatory activity, may contribute positively to the prevention of metabolic and cardiovascular diseases particularly in case honey mixed with other healthy foods. A study conducted on 38 patients and 17 healthy subject consumed natural honey for 30 days has improved the cardiovascular risk factors [122]. Comparing with sucrose, 70 g of daily honey intake has lowered total cholesterol, low-density lipoprotein cholesterol (LDL-C), triglyceride, fasting blood glucose and CRP. Surprisingly, experimental group has recorded a mild reduction in body weight and fat comparing with control group. The previous findings have been explained by [51] where subjects of the study were supplemented with 1.5 g/kg body weight natural honey rich in antioxidant. The plasma phenolics were increased steadily after six hours of honey intake which illustrated the increased antioxidant activity. However, hypercholesterolemia subjects have been given honey for 14 days without improving the serum LDL concentrations [121]. In the previous clinical trial, the subjects have been chosen without differentiating whether they have been taking statins or not. Likewise, the TG, LDL and glucose concentrations have not been changed in impaired glucose intolerant individuals compared with glucose tolerant group after consuming 50 g of honey for two weeks [122]. Exclusively, Dutch Gold honey company has gifted two different clinical trials [51, 123], but their results have been totally controversial. Schramm and his colleagues [51] have confirmed that Dutch gold honey increased the antioxidant activity in healthy subjects while Raatz et al. [123] reported that lipid profile, inflammatory cytokine and insulin sensitivity did not improve in glucose tolerant post-treatment with Dutch Gold honey. The recently previous research has concluded that honey has no effect on improving glycemia and insulin sensitivity including inflammatory cytokines (IL-6). To emphasize, the glycosylated hemoglobin has been increased in diabetic type 2 patients post honey intervention for eight weeks continuously which subsequently could develop cardiovascular diseases [124]. The paradox results of above-mentioned trials may be attributed to various factors: the small number of recruited subjects, type of honey and the duration of intervention. Thus, further clinical trials with controlled conditions should be taken into consideration to facilitate filling the gaps among other scientific reports. In vivo studies using diabetic rats’ model have found that Tualang or Nigerian honey treatment improve the glycemia and dyslipidemia [75]. Based on that, the component of tropical honeys might have high concentration of bioactive compound namely, polyphenols as honeybees expose to a variety of botanical flora as well as the warmth weather. In addition, honey bee has been thought to simulate the characteristics of diverse enzyme inhibitors including, α-glucosidase inhibitors, insulin secretagogues, dipeptidyl peptidase-4 inhibitors and anti-obesity drug [125]. Overall, human and animal studies which are subjected to demonstrate the beneficial effect of honey particularly regarding metabolic and cardiovascular diseases are still insufficient to draw a conclusion.

### Gut microbiota associated with inflammation

A growing body of evidence has indicated that gut microbiome influence strongly not only energy homeostasis but also the inflammatory status in human body. Since the number of gut microbiome is at least 10-fold more than the human body cells, therefore, this large pool of cells provides a significant biological and metabolic function which could not be performed by human cells [126]. The immunological functions of gut microbiota in human body are present by developing and maintaining the mucosal immune system, sheltering against pathogen invasion and preserving the barrier integrity of gastrointestinal tract [127]. In the presence of LPS-producing bacteria in colon along with low levels of beneficial bacteria, gut permeability is increased leading to a higher influx of LPS in plasma, so-called metabolic endotoxemia. In accordance with the previous hypothesis, linking LPS with Toll-like receptor 4 on mononuclear cells may trigger low grade of inflammation which subsequently lead to chronic diseases [128]. There are few supernatural foods that possess a symbiotic characteristic which is a combination of prebiotic carbohydrate with specific probiotic strain [129]. In this context, the prebiotic oligosaccharide and antibacterial compounds existed in honey had been elaborated. Manuka honey has a potentially beneficial efficacy in growing specific types of probiotics (*Lactobacillus reuteri*, *Lactobacillus rhamnosus*, and Bifidobacterium lactis) and inhibiting other pathogens (*Escherichia coli*, Salmonella typhimurium, and *Staphylococcus aureus*). Honey has shown potent probiotic properties when it is incubated, at maximum levels, with milk and/or selective growth media. Thus, pathogenic and intestinal microbes have been inhibited after the treatment [130]. In the light of previous evidences, honey can promote the growth of selective bacteria namely, Lactobacillus and Bifidobacterium genera over other unfavorable microbes. Recently, a new research team has found that encapsulated honey enhances the survivability of Bifidobacterium strains, well-known probiotics, in gastrointestinal simulation comparing with sodium encapsulation [131]. Investigating the probiotic efficacy of honey-rich diet on animal model or human could probably confirm the in vitro results by which understanding the healthy beneficial activity of honey against inflammation is potential. Furthermore, feeding albino rats with two gram of natural honey per day for 6 weeks altered the lactic acid bacteria number to be higher than undesirable microbes [132]. The combination of prebiotic and probiotic (synbiotic) activity of Manuka honey is applied by inhibiting the Helicobacter pylori, a bacterium that cause stomach ulcer, and by supporting the growth of *Lactobacillus reuteri*. More deeply, the two antibacterial components, reuterin from *Lactobacillus reuteri* and methylglyoxal from Manuka honey have a structural similarity [133]. In this prospective, each type of honey probably could support special strain of beneficial bacteria and thus, in vivo, and clinical trials may evaluate and establish the potential prebiotic efficacy of honey oligosaccharide.

## Honey consumption and contradiction

Most often nutritionist and/or physicians tended to alert individuals from consuming high amount of honey and its potential health hazards, however, the ancient usage of honey and the recent discoveries of biological compounds in honey have changed this notice and support the research on its effect on oxidative stress, wound healing, ageing, inflammation, cancer, diabetes, bacterial growth and atherosclerosis. Despite of the mounting evidence of healthy-honey roles, precautions from its consumption are still required. In this sense, honey might contain various of contaminants including pesticides, antibiotics, heavy metals, and other toxic materials which is probably added either through occasional exposure to environmental hazards or through beekeepers to control honeybee maladies. These chemical materials have been proven to cause a seriously unexpected consequence. In addition, the pathogenic contamination with Clostridium botulinum has been considered as a toxic factor, particularly for infants who are below 12 months. For this reason, honey should be sterilized with gamma irradiation to reduce the infectious concern without any loss of natural honey therapeutics [134]. Moreover, the presence of allergens, which is derived from bee glands has been accused to stimulate allergy despite of its rare incidence. However, a case report has been published recently describing that consuming honey and foods-contain honey has caused anaphylaxis [135]. In terms of using honey as topical application, there are various adverse effects that are needed to be controlled. A transient burning sensation has been noticed in wounded patients while diabetes patients may exhibit a high blood glucose along with tissue dehydration. Further, the limited data about honey should be a stimulator for further studies to provide detailed results about the exact content of bioactive compounds, particularly polyphenols. More deeply, the above mentioned have mainly attributed the healthy benefits of honey to its polyphenols content. Therefore, honey-producers are encouraged to label the phenolic content on their honey products.

## Conclusion

**Fig. 3:**
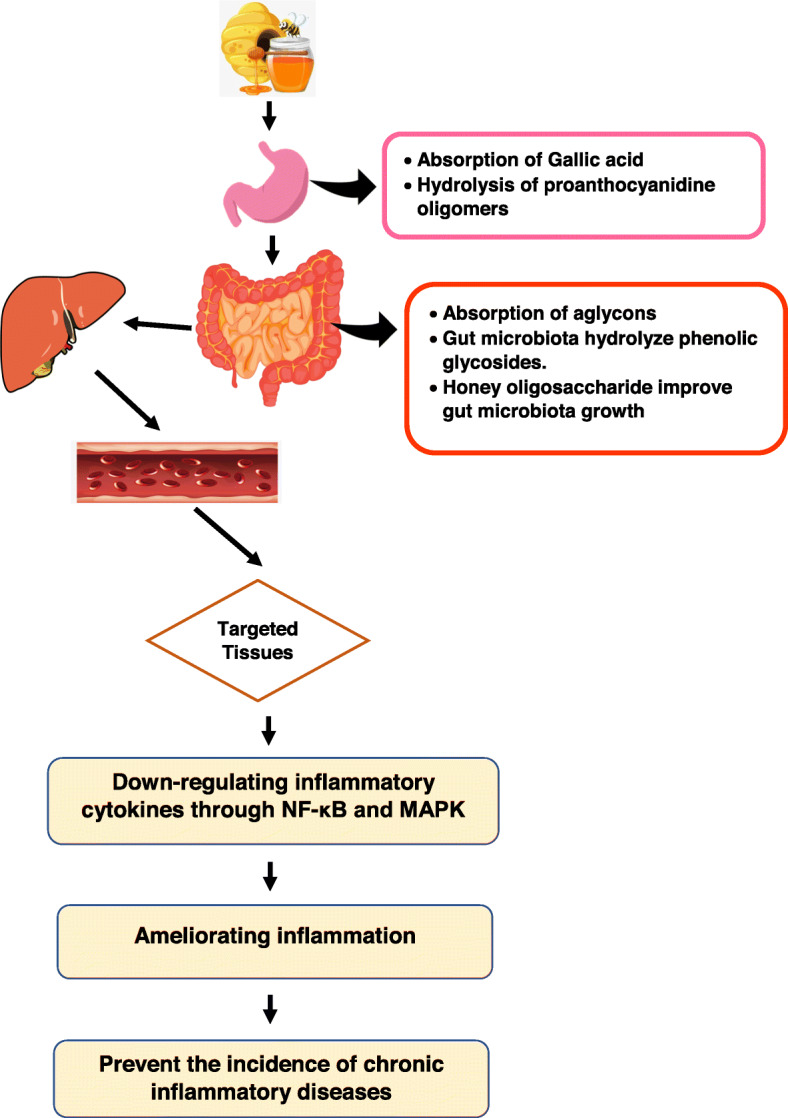
The absorption of honey-bioactive compounds and the proposed mechanism of their therapeutic actions against inflammation

Honey consumption has high nutritional and therapeutic values. The phytochemical compounds in honey depends mainly on various factors, floral source, honey type, concentration, and bee type. These factors affect the biological activities of each types of honey. Basically, most therapeutic properties of honey come from the polyphenols. The aforementioned studies confirmed that honey polyphenols have high bioavailability value compared to other functional foods due to various reasons; 1) lack of food matrix in honey composition, 2) lack of food interactions, and 3) honey polyphenols are not exposed to any chemical reaction such as extraction or decoction which could affect their quality. Thus, targeted tissues benefit from the honey’s pharmacological and preventive actions which modulate the inflammatory cytokines functions and ultimately reduce the severity of chronic inflammatory diseases (Fig. 3). However, the molecular mechanisms of polyphenols rich in honey are not fully illustrated. For this reason, further investigations are still needed in the nutrigenomics analysis to fully elucidate the genome-wide influences of honey and patterns of global gene expression, protein expression, intracellular signaling pathways, and metabolite production in response to particular compounds. Finally, examine the effects of honey on the gene expression profiling with special emphasis on human intervention trials, ideally with large-scale randomized placebo-controlled studies, in order to yield important insights into their prophylactic and therapeutic uses, as well as develop effective strategies for alleviating chronic inflammatory diseases.

## Data Availability

The datasets during and/or analysed during the current study is available from the corresponding author on request.

## Abbreviations

ROS: Reactive oxygen species
LDL: Low density lipoprotein
MAPK: Mitogen activated protein kinase
NSAIDS: Nonsteroidal anti-inflammatory drugs
ERK: Extracellular signal regulated kinase
RAS: Reticular activating system
HT-29: *Homo sapiens* colorectal adenocarcinoma cell line
NFKB: Nuclear factor kappa light chain enhancer of activated B cells
GI: Gastrointestinal
IKBα: Inhibitor of nuclear factor kappa B alpha
IKK: IKB kinase enzyme complex
IL: Interleukin
COX: Cyclooxygenase
PGE: Prostaglandin E
NO: Nitric oxide
